# The Grapevine Microbiome to the Rescue: Implications for the Biocontrol of Trunk Diseases

**DOI:** 10.3390/plants11070840

**Published:** 2022-03-22

**Authors:** Rebeca Cobos, Ana Ibañez, Alba Diez-Galán, Carla Calvo-Peña, Seyedehtannaz Ghoreshizadeh, Juan José R. Coque

**Affiliations:** 1 Instituto de Investigación de la Viña y el Vino, Escuela de Ingeniería Agraria, Universidad de León, 24009 León, Spain; rebeca.cobos@unileon.es (R.C.); aibas@unileon.es (A.I.); aldig@unileon.es (A.D.-G.); ccalp@unileon.es (C.C.-P.); tannaz_gh26@yahoo.com (S.G.); 2 Área de Microbiología, Departamento de Biología Molecular, Universidad de León, 24071 León, Spain

**Keywords:** biocontrol agents, grapevine microbiome, grapevine mycobiome, grapevine trunk diseases, metagenomics

## Abstract

Grapevine trunk diseases (GTDs) are one of the most devastating pathologies that threaten the survival and profitability of vineyards around the world. Progressive banning of chemical pesticides and their withdrawal from the market has increased interest in the development of effective biocontrol agents (BCAs) for GTD treatment. In recent years, considerable progress has been made regarding the characterization of the grapevine microbiome, including the aerial part microbiome (flowers, berries and leaves), the wood microbiome, the root environment and vineyard soil microbiomes. In this work, we review these advances especially in relation to the etiology and the understanding of the composition of microbial populations in plants affected by GTDs. We also discuss how the grapevine microbiome is becoming a source for the isolation and characterization of new, more promising BCAs that, in the near future, could become effective tools for controlling these pathologies.

## 1. Introduction

Grapevine trunk diseases (GTDs) are widely recognized as a major threat to the sustainability of the grape industry and the wine sector worldwide, since they severely compromise the longevity and productivity of grapevines (*Vitis vinifera* L.), causing severe annual economic losses by increasing production costs [1].

**Figure 1:**
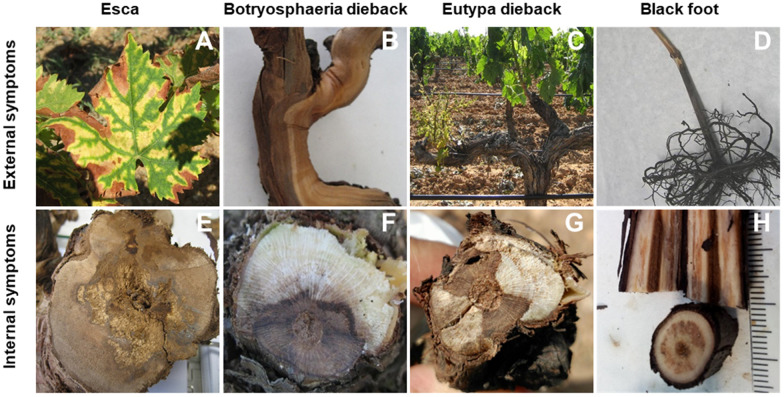
Typical external (**A**–**D**) and internal (**E**–**H**) symptoms that can be observed in plants affected by some of the most representative GTDs (esca, Botyrosphaeria dieback, Eutypa dieback and black-foot disease).

Several factors contribute to the enormous difficulty of controlling GTDs. First of all, the term “trunk diseases” encompasses a wide range of pathologies and syndromes, among which some of the most representative are black-foot disease, Botryosphaeria dieback, Eutypa dieback, esca, excoriose and Petri disease or young grapevine decline. Consequently, the number of pathogenic fungi (mainly ascomycetes and basidiomycete fungi) associated with GTDs is very large. In fact, as of 2018, as many as 133 fungal species belonging to 34 genera had been associated with GTDs worldwide [1], and this number increases every year. These fungal pathogens produce a wide variety of external and internal symptoms [1,2,3,4,5,6,7] (Figure 1). External symptoms are varied, depending on the pathology analyzed. Among them, we can mention as the most typical the “tiger-stripe” patterns observed in the leaves of some esca-affected plants (Figure 1A), the longitudinal canker-like lesions appearing on the arms and trunk of plants affected by Botryosphaeria dieback (Figure 1B), the appearance of stunted shoots with chlorotic leaves and bunches that ripen unevenly (Figure 1C) in plants affected by Eutypa dieback or the significant reduction in root biomass (Figure 1D) observed in plants affected by black-foot disease. The main internal symptoms can vary from internal necrotic lesions observed in the cross-sections of branches and trunks that involve different degrees of wood affectation (Figure 1E–G) or that manifest as a black discoloration and necrosis of wood tissues, which develop from the base of the rootstock in plants affected by black-foot disease (Figure 1H).

With such a high number of fungal pathogens involved, the pathological processes and mechanisms of infection are expected to be very varied. In fact, these fungi can infect the plant by using different routes of entry, and this further complicates the overall situation. Indeed, the infection of adult plants in mature vineyards can occur through the root system. This is the primary mode of colonization for pathogens belonging to the *Dactylonectria* or *Ilyonectria* genera, among others [2,3], which are involved in black-foot disease. Other pathogens, such as species of the *Botryosphaeriaceae* family and *Eutypa lata*, primarily penetrate the plant through pruning wounds that are produced at the end of the growing season each year [4,5]. Finally, some pathogens, such as *Phaeoacremonium minimum* or *Phaeomoniella chlamydospora*, can use both pathways to penetrate the plant, and these two microorganisms are the primary causal agents of Petri disease [2,6]. Young grapevine plants can be infected in the field by the same mechanisms described for adult plants. However, it is well-known and established that the main source of infection for young plants is the infection of planting material (grafted plants) produced in nurseries. In fact, it has been widely reported that grafts can be infected at different stages of the propagation process that takes place in nurseries [2,3,6]. Given the great negative impact of GTDs in the wine sector, it should come as no surprise that, over the last 25 years, enormous efforts have been made to develop chemical control methods (both pesticides and natural products) or biocontrol agents (BCAs), as reviewed by Mondello et al. [7], to fight these pathologies. However, in many developed countries, including those of the European Union, policies have been implemented in recent years to ban and/or limit the use of pesticides for crop pest control (e.g., the European Green Deal initiative, one of whose objectives is to limit the use of synthetic chemical pesticides in the agri-food sector as much as possible by 2030). As a result, there are currently no chemicals available on the market with a proven efficacy against GTDs.

Unfortunately, and independently of their mode of action, one major difficulty associated with their use, for both chemical pesticides and BCAs, is their inconsistent performance in the field. Moreover, many of the commercially available BCAs have not been isolated from the plant/organ that they are intended to protect, and this could have a significant influence on their inconsistent efficacy and survival in the field [8]. Thus, it is not uncommon to find commercial BCAs isolated from the rhizosphere that, surprisingly, are used as grapevine foliar sprays or on pruning wounds; however, these habitats are quite different, and colonizing microorganisms, which may be highly adapted to one habitat (rhizosphere), could be totally unable to thrive in another habitat (phyllosphere or pruning wounds) of the same plant [8]. Thus, to date, none of the BCAs tested has been proven to be effective against all GTD-causing pathogens, perhaps because many of the BCAs more frequently used to fight GTDs are *Trichoderma* strains that were isolated in the 1990s from many different origins (not necessarily even vineyard ecosystems), and therefore may not be optimally adapted to the crop. 

In this context, in recent years, researchers have been showing increasing interest in characterizing the grapevine microbiome as a possible source of more efficient BCAs, well-adapted to the ecological niche where they should be applied and also adapted to coexist with and fight against a specific pathogen. In this manuscript, we review the most recent advances in the analysis of the grapevine microbiome and how this could result in the development of more efficient BCAs for GTD control.

## 2. Insights into the Grapevine Microbiome: The Microbiome Concept

*Vitis vinifera* is one of the most cultivated fruit crops worldwide, with a great economic impact on the economy of many countries. As in any other crop, many different associated microorganisms colonize both the exterior (epiphytes) and interior surfaces (endophytes) of grapevines. These associated microbiota play key roles in many different aspects, such as plant health (stimulating plant defense mechanisms or inhibiting phytopathogens) and productivity or mineralization of soil organic matter [9,10]. It should come as no surprise, therefore, that characterization of the grapevine plant microbiome has gained growing interest in recent years, in an effort to elucidate the impact that all of these microorganism–plant relationships could have on the crop.

**Figure 2:**
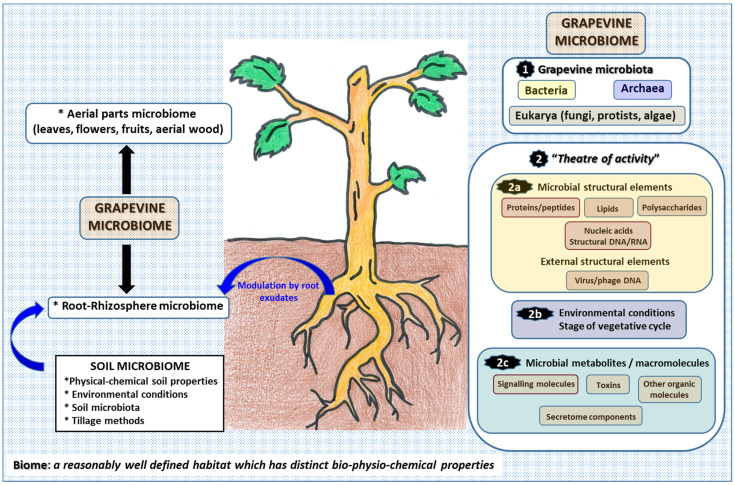
Schematic representation of the grapevine microbiome concept based on the modern meaning of microbiome established by Berg and colleagues [11] that includes both the microbiota that inhabits grapevine plants and the so-called “theatre of activity” concept that includes microbial structural elements, microbial metabolites and macromolecules, and the surrounding environmental conditions or the stage of vegetative cycle that greatly influence the microbial development.

According to a modern review of the microbiome concept, the microbiome is defined as follows: “a characteristic microbial community occupying a reasonable well-defined habitat which has distinct physio-chemical properties. The microbiome not only refers to the microorganisms involved but also encompasses their “theatre of activity”, which results in the formation of specific ecological niches. The microbiome, which forms a dynamic and interactive micro-ecosystem prone to change in time and scale, is integrated in macro-ecosystems including eukaryotic hosts, and here crucial for their functioning and health” [11]. This definition, therefore, considers the role of the so-called “theatre of activity” to be crucial for understanding the microbiome as a whole. This “theatre of activity” would include microbial structural elements (proteins/peptides, lipids and polysaccharides, nucleic acids and mobile genetics elements), microbial metabolites (signaling molecules, toxins and organic molecules) and the surrounding environmental conditions (Figure 2).

Next, we review some attempts to describe and define the grapevine microbiome (Table 1) and how it can impact crop quality. Special attention is given to the microbiome in relation to the presence of fungal pathogens involved in GTDs, and also as a valuable source of possible microorganisms or metabolites of interest, for the control of GTDs.

## 3. The Microbiome of Aerial Parts (Leaves, Flowers and Berries)

In recent years, several studies have analyzed the microbiome of aerial parts of the grapevine plant, other than perennial wood, such as leaves, flowers and berries, as we describe below.

Grapevine leaves are frequently used as biomarkers for the phytosanitary status of the plant. They are the organ with the greatest surface area of the plant. They are also abundant, and if we compare them to other structures, such as fruits or flowers, which are not always present, leaves are more permanent structures, thus allowing for an analysis of most of the plant’s vegetative cycle. Moreover, leaves are more convenient to sample than wood, while processing is more complicated and requires producing cuts that could compromise the vitality of the plant [12]. Pinto and colleagues [12] analyzed the microbiome of grapevine leaf samples collected from a vineyard (cv. Tempranillo) located in the Barriada appellation (Cantanhede, Portugal) (Table 1) in order to characterize the evolution of microbial communities during the vineyard’s vegetative cycle. Bacterial communities at the phylum level were primarily composed of Proteobacteria, (31.2%), Firmicutes (29.4%) and Actinobacteria (19.4%), with a lower proportion of Acidobacteria and Bacteroidetes. At the family level, the bacterial community was dominated by members of *Streptococcaceae, Enterobacteriaceae, Pseudomonadaceae and Moraxellaceae*. 

Similar results were reported by Zarraonaindia and colleagues [13], who analyzed the composition of bacterial communities in aboveground samples (leaves, grapes and flowers) of five different vineyards (Merlot cultivar) in Long Island (NY, USA) (Table 1). All aboveground samples were largely composed of Proteobacteria (grapes, 80.7%; leaves, 90.0%; and flowers, 98.0%), whereas Firmicutes, Acidobacteria and Bacteroidetes were detected in low abundance. In leaf and grape samples, Proteobacteria from *Pseudomonas* (leaves, 43%; grapes, 19%), *Sphingomonas* (leaves, 19%; grapes, 33%) and *Methylobacterium* were the most abundant genera. Meanwhile, in flower samples, *Pseudomonas* (61.8%) and *Erwinia* (25.2%) were the dominant Proteobacteria taxa.

The bacterial leaf microbiomes from Pinot Noir, Chasselas and Solaris varieties (Prangins, Switzerland) were analyzed by characterizing epiphytic and endophytic communities [8,14], although using a classical culture-dependent methodology. A total of 194 bacterial strains were isolated, and a much greater diversity was observed in the epiphytic communities (12 genera) than in the endophytic ones (six genera). A strong dominance of *Staphylococcus* and *Bacillus* isolates was observed in all varieties, especially among the endophytes. Only two Gram-negative isolates (belonging to *Erwinia* and *Sphingomonas* genera) were detected among the total isolated endophytes. However, a higher diversity of Gram-negative strains was detected in the epiphytic community. 

Bacterial diversity analyses of Grenache and Carignan cultivar berries sampled in the Priorat region (Tarragona, Spain) (Table 1) showed that bacterial communities were different at each of the five vineyards analyzed; grape variety, geographical location and orientation were all related to changes in bacterial populations [15]. Up to 14 bacterial phyla were detected, with Firmicutes (37.6% Bacillales and 14% Lactobacillales), Proteobacteria (16.8% Pseudomonadales and 11.6% Enterobacteriales) and Actinobacteria (3.4% Actinomycetales) being the most abundant.

**Table 1:** More relevant studies analyzing the grapevine plant microbiome or the vineyard soil microbiome by using next-generation sequencing (NGS) or metatranscriptomic approaches.

Tissue/Material Analyzed	Grapevine/Rootstock Variety *	Geographical Area	Analysis Type	Microbiome Analyzed ^#^	Year/Reference
Leaves	Tempranillo	Cantanhede, Portugal	Pyrosequencing	B, F	2014 [12]
Leaves, flowers, grapes, roots, rhizosphere, bulk soil	Merlot	Long Island, NY, USA	Illumina NGS	B	2015 [13]
Grapes	Carignan, Grenache	Priorat region, Tarragona, Spain	Illumina NGS	B	2016 [15]
Grapes	Corvina	Gargagnago di Sant’Ambrogio di Valpolicella, Italy	Illumina NGS	B, F	2016 [16]
Bulk soil	ND	Napa Valley, CA, USA	Illumina NGS	B	2016 [17]
Bulk soil	ND	Central Chile	Roche 454 Gs Junior Titanium Series	B, F	2017 [18]
Bulk soil	Cabernet Franc	Trentino South Tyrol (Italy)	Roche Gs FLX+ system	B, F	2017 [19]
Bulk soil, rhizosphere	Pinot Noir	Carpeneto, Italy	Roche 454 Pyros.	B	2017 [20]
Bulk soil, rhizosphere	Zweigelt grafted on BB5	Lake Neusiedl, Austria	Illumina NGS	B	2017 [21]
Grapes, bulk soil	Riesling	Ovid, NY, USA	Illumina NGS	B, F	2018 [22]
Bulk soil, rhizosphere, root endosphere	Lambrusco grafted on 5BB, 1103P	Finale Emilia, Modena, Italy	Illumina NGS	B	2018 [23]
Bulk soil	Riesling	Geinsenheim, Germany	Illumina NGS	B, F	2018 [24]
Shoots, leaves, flowers, bark, root	Red Globe, Cabernet Gernischt	Beijing and Yunnan (China)	Illumina NGS	F	2018 [25]
Wood	Midnight beauty	Beijing (China)	Illumina NGS	F	2018 [26]
Wood	Pinot Meunier	Sonoma county (CA, USA)	Metatranscriptomic	F	2018 [27]
Wood	Sauvignon Blanc, Grenache	Czech Republic, Spain	Metatranscriptomic	F	2018 [28]
Rhizosphere, roots	Barbera grafted on 402A, 157.11, SO4, 161.49C	Oltrepo Pavese (Italy)	Illumina NGS	B	2018 [29]
Wood	Cabernet Sauvignon	Lisbon (Portugal)	Illumina NGS	F	2019 [30]
Rhizosphere	110R, 140Ru, 1103P, 41B, 161-49C	Aldeanueva de Ebro and Olite (Spain)	Illumina NGS	B, F	2019 [31]
Bulk soil, rhizosphere, endorhizosphere	Tempranillo	Las Rioja (Spain)	Illumina NGS	F	2019 [32]
Wood	Verdelho, Shiraz	Hilltops and Hunter Valley. New South Wales (Australia)	Illumina NGS	B, F	2020 [33]
Rhizosphere	1103P, 140 Ru, 161-49 C, Kober 5BB	Rheingau, Germany	Ion Torrent Seq.	B	2021 [34]
Bulk soil, rhizosphere, root, cordon, cane, sap	Syrah	Temecula (CA, USA)	Illumina NGS	B, F	2020 [35]
Wood	Xinomavro, Agiorgitiko, Vidiano	NW (Amyntaio), central-south (Nemea) and southern Greece (Crete)	Illumina NGS	B, F	2021 [36]
Wood	Malbec	Luján de Cuyo (Argentina)	Metatranscriptomic	B, F	2021 [37]
Wood	16 varieties grafted on 4 rootstocks	Cataluña (Spain)	Illumina NGS	F	2022 [38]

* Not determined (ND); Couderc 161-49 (161-49C); Kober 5BB (5BB); Paulsen 1103 (1103P); Richter 110 (110 R); Ruggeri 140 (140Ru); 41B Millardet et de Grasset (41B); 420A Millardet et de Grasset (420A); ^#^ bacterial microbiome (B); fungal microbiome (F).

Salvetti and colleagues [16] analyzed the bacterial microbiome of Corvina berries (Sant’Ambrogio di Valpolicella, Italy) (Table 1) under two different withering conditions (traditional withering (TW) or accelerated withering (AW)). A total of 25 bacterial phyla were detected at the end of the drying process, nine of which were on the berry surfaces from both types of samples analyzed. Proteobacteria was the predominant phylum in both samples (with levels ranging between 86.1 and 97.7%), and the less abundant phyla included Firmicutes (7.8%), Bacteroidetes (ranging from 1.2 to 4.7%) and Actinobacteria (0.7%). 

In general, all of these studies show that the bacterial microbiota composition of plant aerial parts is strongly conditioned by many different factors, including the different stages of the vegetative cycle [12], grape variety, geographical location, vineyard orientation [13,15] or composition of the soil microbiota, so that many of the constituent individuals can colonize the aboveground plant organs. This suggests that the vineyard soil can act as a reservoir from which microbes can colonize the phyllosphere [13]. However, in a different study that was carried out in vineyards located in Ovid (NY, USA), Chou and colleagues [22] concluded that, although vineyard under-vine floor management had a deep impact on the soil microbial composition, the grapes did not show significant variations in their microbial populations in a similar way. 

The eukaryotic leaf community composition was analyzed by Pinto and colleagues (Table 1) [12]. The leaf eukaryotic microbiome was characterized by a predominance of early divergent fungal lineages (27.9%) and members of Ascomycota (26.3%) and Basidiomycota (16.9%) phyla. The most detected genera were *Rhizopus*, *Mucor* and the entomopathogens *Zoophthora* and *Pandora* (all belonging to the early diverging fungal lineage group). The most frequently detected ascomycetes belonged to the *Aureobasidium*, *Sporormiella* and *Alternaria* genera, whereas *Kurtzmanomyces*, *Colacoglaea*, *Ustilago*, *Puccinia* and *Cronartium* were the more abundant of the Basidiomycetes.

The composition of fungal communities developing on Riesling grapes (Ovid, NY, USA) (Table 1) indicated a predominance of *Sporobolomyces*, *Aureobasidium*, *Rhodosporidium*, *Penicillium* and *Entyloma*, although relative abundances of these five predominant genera over the 3 years of the study were widely variable [22]. 

In Corvina berries dehydrated under two post-harvest conditions [16], the eukaryotic community was primarily composed of Ascomycetes belonging to the Eurotiomycetes class (*Aspergillus* and *Penicillium* genera being the most represented), but also to the classes Sordariomycetes (mainly *Gibberella* and *Neurospora* representatives) and Dothideomycetes (specifically the *Phaeosphaeria* and *Pyrenophora* genera). Although it seems logical to think that most of these microorganisms are already present in the grapes at source, it is no less evident that the drying process should have a strong impact on the composition of the dominant microbial populations after drying, so it is not possible to establish comparisons with the populations present in grapes directly sampled from the plant.

In general, studies of the leaf, flower and fruit microbiomes have not allowed for detection, at least among the most frequently represented genera, of any of the organisms responsible for GTDs; therefore, it can be assumed that fungi causing GTDs are not normal inhabitants of these plant aerial environments [5].

## 4. The Endomicrobiome and Wood Microbiome

Until a decade ago, the characterization of the complex microbial communities that inhabit grapevine wood, either as saprophytes or endophytes, consisted of culture-dependent studies, in which microorganisms were isolated in vitro and later identified by a combination of both morphological traits and molecular techniques. Thus, although the number of papers that have addressed the isolation and characterization of saprophytic and endophytic mycobiota from grapevine wood is very large (for a comprehensive review, see Jayawardena et al. [25]), the studies that have used a next-generation sequencing (NGS) approach to characterize the microbial populations inhabiting grapevine wood of the aerial parts are scarce, as we review next.

### 4.1. The Microbiome of Grapevine Wood Endophytic Bacteria 

In contrast to the endophytic mycobiome, the characterization of endophytic bacterial populations by using NGS strategies has received less attention. In a work cited above, Niem and colleagues [33] analyzed the composition of endophytic bacteria in symptomatic and asymptomatic plants (Table 1). When considering abundance at the phylum and genus level, in all samples, the dominant phylum among the prokaryotic community was Proteobacteria, accounting for 51–96% of the total reads for the given taxa, followed by Actinobacteria with 3–44%. The Bacteroidetes, Firmicutes and Chloroflexi phyla represented less than 10% of the OTUs detected. At the genus level, with the exception of symptomatic samples from the Verdelho variety, the most abundant genus within the Proteobacteria phylum was *Pseudomonas* (ranging from 29 to 74%). It is also noteworthy that, in both locations, *Pseudomonas* had a much higher relative abundance in asymptomatic samples for GTDs as compared to samples from symptomatic vines. This could indicate its ability to colonize and survive in the interior of grapevine. More interestingly, several *Pseudomonas* endophytic isolates exhibiting important antifungal activity against different GTD-associated pathogens were obtained [33]. 

The wood bacterial microbiome from symptomatic and asymptomatic plants has also been analyzed in different Greek vineyards (Table 1). It was composed of 30 phyla. Proteobacteria (mean relative abundance 49.5%) was the dominant phylum, followed by Actinobacteriota (17.6%), Bacteroidota (13.4%), Firmicutes (9.3%), Planctomycetota (1.9%), Abditibacteriota (1.4%), Verrucomicrobiota (1.4%), Acidobacteriota (1.1%) and Chloroflexi (0.8%), totaling a relative abundance of 94.4–99.3%. The bacterial microbiome in asymptomatic plants was dominated by *Bacillus* and *Streptomyces*, which showed a negative co-occurrence pattern with *Phaeomoniella*, *Phaeoacremonium* and *Seimatosporium*. These data could suggest a putative role of these bacterial taxa in the suppression of GTDs [36].

More recently, the endophytic bacterial microbiome of different samples (root, cordon, cane and sap) was analyzed at a Syrah vineyard in California (USA) (Table 1) [35]. In every area, Proteobacteria was the dominant phylum (ranging from ~80% of relative abundance of the cane bacteriome to <40% in sap samples), except in the sap compartment, where Firmicutes (39.7%) were more abundant. This phylum and Actinobacteria were also presented in all the niches at relative abundances greater than 10% of average. Seven taxa emerged as dominant as they inhabited all the biocompartments of the grapevine endosphere: *Escherichia*/*Shigella*, *Novosphingobium*, *Pseudomonas*, *Rhizobium*, *Sphingomonas*, *Bacillus* and *Steroidobacter*). *Streptomyces* was also an abundant taxa commonly found in all the vine lignified tissues (root, cordon and cane) and could also be found frequently in the sap [35].

### 4.2. The Endophytic Mycobiome of Grapevine Wood of Aerial Parts

The characterization of Eukaryotic endophytes in grapevine stems (cv. Midnight Beauty) from plants of three different ages (3, 8 and 13 years old) sampled in Beijing (China) (Table 1) was carried out by using both a culture-dependent and a high-resolution culture-independent NGS approach [26]. In the culture-dependent approach, a total of 94 isolates belonging to 19 genera were isolated, of which *Alternaria*, *Cladosporium* (belonging to the Dothideomycetes class) and *Aspergillus* (Eurotiomycetes class) were the most abundant (with a relative abundance of 28.7%, 21.1% and 10.6%, respectively). Remarkably, two fungal species associated with GTDs were detected, namely *Botryosphaeria dothidea* (5.3%) and *Lasiodiplodia theobromae* (1.1%), which are members of the *Botryosphaeriaceae* family involved in the “black dead arm” syndrome, also known as *Botryosphaeria* dieback [1,5]. The diversity of the fungal community detected by using an NGS approach was higher, since 59 operational taxonomic units (OTUs) were detected. The richness of endophytic fungi increased with plant age and was highest for the 13-year-old plants. The fungi identified belonged primarily to three phyla: Ascomycota (93.6%), Basidiomycota (4.2%) and Zygomycota (2.1%). Whereas most (51 out of 59) of the fungal OTUs detected were infrequent (they had a relative abundance lower than 0.1%), the most frequently detected fungi belonged to the *Cladosporium* genus (52.13%), Ascomycota group (11.85%) and, remarkably, the putative pathogens *Cadophora* (i.e., *C. luteo-olivacea*) (1.60%) and *Botryosphaeria* (0.45%) involved in GTDs. Remarkably, the comparison of fungal taxa identified by both approaches resulted in an overlap of 53% of fungal genera, despite the fact that, due to the interspecific variability of the sequences obtained from NGS, the OTUs were only assignable to the order, family or genus level, thus constituting a significant limitation of the NGS technique used.

A similar study was also carried out in China with samples of decaying plants from the Red Globe table grape variety and the Cabernet Gernischt wine variety (Table 1) [25]. A traditional culture-dependent strategy allowed for the detection of 45 taxa (belonging to 30 genera) of which 19 were detected in both cultivars. From the identified isolates, 32.6% were Sordariomycetes, 26.1% Dothideomycetes 19.7% Eurotiomycetes, 6.5% Mucoromycetes, 4.4% Agaricomycetes, 2.2% Leotiomycetes and 2.2% *Oomycota incertae sedis*. In the two analyzed plant varieties, several GTD-causing fungi were detected among these 45 taxa, including *B. dothidea* and *Dothiorella sarmentorum* involved in Botryosphaeria dieback [1,5]; *Neopestalotiopsis vitis*, which is associated with both GTDs and grapevine leaf spot [39]; and *Diaporthe eres* [40]. The culture-independent approach, based on Illumina^®^ sequencing targeting the Internal Transcribed Spacer ITS1, yielded a greater fungal diversity: 226 OTUs were detected, with 176 and 189 belonging to the Cabernet Gernischt and Red Globe cultivars, respectively. There were 139 fungal OTUs shared between the two cultivars and 37 and 50 OTUs were specific to the Cabernet Gernischt and Red Globe cultivars, respectively. In Cabernet Gernischt, members of Ascomycota phylum were dominant, accounting for 77% (mainly 46% Sordariomycetes, 19% Eurotiomycetes and 7% Dothideomycetes) of the total sequences, followed by an unidentified phylum (23%) and Basidiomycota and Zygomycota (less than 0.1%). In Red Globe, almost all sequences (97%) were assigned to Ascomycota (51% Eurotiomycetes, 42% Sordariomycetes and 3% Dothideomycetes), with a smaller proportion of Basidiomycota (3%). Analysis of the top 20 taxa detected in both cultivars allowed for the detection of different fungi associated with GTDs, including *Ilyonectria macrodidyma* (7.2–7.9%), one of the most important pathogens associated with black foot disease [1,2,3], and members of *Nectriaceae* (5.6–5.77%) and *Diaporthaceae* (7.04–7.72%) families [25]. Both studies indicated that traditional culture-dependent techniques are clearly more suitable methods for accurately identifying taxa. However, culture-independent methods can provide a more complex, complete and better understanding of the entire microbial population present in a host in a natural environment.

More recently, an Illumina^®^ NGS approach was used to characterize the wood mycobiome in a vineyard (c.v. Cabernet Sauvignon; Lisbon, Portugal) affected by esca (Table 1) [30]. Samples were taken from both symptomatic (with leaves exhibiting the typical tiger stripe symptoms of esca) and asymptomatic plants in order to understand the putative existence of a link between microbial communities in the woody parts and expression of esca leaf symptoms. A total of 289 taxa could be assigned to genus or species level, and 50 of them were found in relative abundance greater than 0.1%. The fungal community detected in perennial wood was dominated by the presence of ascomycetes and basidiomycetes (66.7% and 26.7%), with a high abundance of the tracheomycotic pathogen *P. chlamydospora* (25.8%) and the white-rot agent *Fomitiporia* sp. (14.6%), two organisms directly associated with esca proper and other esca-related syndromes, such as the wood decay observed in advanced stages of the pathology [1]. Other wood-decaying agents, such as *F. mediterranea* (0.2%) and *Inonotus hispidus* (0.3%) [41], were also identified in this study, along with several others represented in minor abundance (e.g., *Fomitiporella* sp.). Other GTD pathogens detected among the 30 most abundant taxa were *E. lata* (0.7%) and *Eutypa leptoplaca* (0.9%), within the *Diatrypaceae,* pathogens involved in Eutypa dieback [1,5]. Other members of this family detected, also involved in Eutypa dieback, were *E. flavovirens*, *Eutypella citricola* and *Cryptovalsa ampelina* [1,5]; however, they were identified as rare taxa (relative abundance lower than 0.1%). Pathogens of *Nectriaceae* (*Ilyonectria* sp. and *Neonectria* sp.) and also *Botryosphaeriaceae* families, such as *Diplodia pseudoseriata*, *Neofusicoccum parvum* and *Neofusicoccum austral* [1,2,5], were also found; however, they were also represented as rare taxa. Interestingly, the mycobiome of the woody tissue in the proximity of leaves exhibiting the typical tiger stripe symptoms of esca was not significantly different from that of plants not exhibiting any leaf symptomatology (according to the Shannon diversity Index) [30]. 

The endophytic mycobiome of symptomatic and asymptomatic plants of Verdelho and Shiraz vines was analyzed in vineyards from two areas located in New South Wales (Australia) (Table 1). The mycobiome was mostly characterized by a preponderance of Ascomycota (ranging between 83 and 99% relative abundance) from both asymptomatic and symptomatic samples at both vineyards. Remarkably, at the genus level, the fungal community was dominated by the GTD-associated pathogens *Phaeomoniella* (primarily *P. chlamydospora*), representing 59–89% of the total fungi detected, and *Phaeoacremonium* species, specifically *Ph. iranianum* (5–21%), except for a divergence in asymptomatic vines from the Hunter Valley (one of the two geographic areas under study), where *Inonotus*, from the phylum Basidiomycota, came in second at 18% relative abundance [33].

In a more recent work Lade and colleagues [38] analyzed and compared necrosis and fungal communities in graft unions and root collars in combinations of eleven red and five white grapevine varieties with four common rootstocks coming from six nurseries in Catalonia (Spain) (Table 1). A total of 732 OTUs were detected. Of these, 61 OTUs were identified at the genus level, 11 of which were considered putative pathogens involved in GTDs (*Botryosphaeria*, *Cadophora*, *Campylocarpon*, *Cylindrocarpon*, *Diplodia*, *Eucasphaeria*, *Lasiodiplodia*, *Neofusicoccum*, *Phaeoacremonium*, *Phaeomoniella* and *Phomopsis)*. When fungi were isolated from graft unions and root collars, the isolated GTD-associated pathogens were *Dactylonectria* sp., *D. alcacerensis*, *D. anthuriicola*, *Diaporthe* sp., *Diaporthe celeris*, *Diaporthe hispaniae*, *Ilyonectria* sp., *I. liriodendra*, *Pestalotiopsis* sp. and *Thelonectria*. These pathogens differed from those identified in the metagenomics analysis, except for *Botryosphaeria* sp., *Diplodia* sp. and *N. parvum*, which were detected in both analyses. Again, this study highlights the importance of the methodology used to identify fungi. These techniques rendered two distinct sets of GTD-related fungi in young nursery plants with little crossover: those that were determined from sequencing isolates collected from symptomatic tissues and those that could only be detected with NGS technology. This work has also led the researchers to conclude that nursery origin, followed by rootstock type and variety, had significant effects on necrosis and fungal community structure in graft and root tissues. Within the plant, they found large differences in terms of fungal community distribution between graft and root tissues. Graft unions housed a significantly higher relative abundance (48.6%) of GTD-related OTUs than root collars (7.2%), and, interestingly, *C. luteo-olivacea* was the most represented GTD-associated pathogen in both tissues. Another interesting finding of this work was that more severe necrosis was correlated with a lower relative abundance of GTD-related OTUs. This finding is intriguing, since it could suggest that other factors, not yet identified and different from the composition of the community of GTD-associated pathogens, could be involved in the development of necrosis. We must remember that a previous work also indicated similarities in the community structure of symptomatic and non-symptomatic woody tissues [30], although no consistent explanation has been given as to why some communities induce necrosis, and others do not. 

Fotios and colleagues [36] analyzed the wood mycobiome from symptomatic and asymptomatic vines in different Greek vineyards (Table 1). Overall, the fungal wood microbiome was composed of Ascomycota (mean 88.7%) and Basidiomycota (mean 10.8%) as most abundant phyla. In total, 31 fungal classes were identified with the most abundant being Dothideomycetes (46.2%), Sordariomycetes (21.8%), Eurotiomycetes (13%), Agaricomycetes (8.1%), Leotiomycetes (4.6%) and Lecanoromycetes (2%), summing to a total relative abundance of 95.7%. *P. chlamydospora*, *Kalmusia variispora*, *Fomitiporia* spp. and *Diaporthe* spp., all previously proposed as causal agents of GTDs, along with *Acremonium* sp. (non GTD-linked fungus), were consistently associated with symptomatic vines in a cultivar-dependent manner [36].

### 4.3. The Endophytic Mycobiome of Grapevine Wood According to Metatranscriptomic Approaches

Most of the studies characterizing the grapevine wood mycobiome, as we have just reviewed above, were carried out by using ribosomal DNA gene sequencing, which provides a description of all members of the community regardless of their activity level, in such a way that it is not possible to distinguish between dead or viable microorganisms with intact genetic material. However, sequencing of ribosomal DNA transcripts (rRNA) can reveal the metabolically active fungal taxa, providing insights into their activity in environmental samples [28].

This approach has been recently used by Morales-Cruz and colleagues [27] to analyze the microbiome of grapevine wood from plants affected by trunk diseases in different California grape production areas (Table 1). Wood samples were used for DNA (classical metagenomics DNA-Seq analysis) and RNA isolation (metatranscriptomic RNA-Seq analysis). The same samples were also used for culture-dependent identification. This last strategy led to the identification of *E. lata* isolated from all the plants exhibiting foliar symptoms, whereas *P. chlamydospora* and *Ph. minimum* were recovered from 50% and 38%, respectively, of vines with esca symptoms. Other pathogens isolated from vines with no foliar symptoms were *P. chlamydospora*, *N. parvum*, *D. seriata* and *Diaporthe ampelina*. The combination of metagenomics and metatranscriptomic approaches allowed investigators to identify *E. lata* (83.05%) as the predominant species in samples with Eutypa dieback symptoms, with *P. chlamydospora* (7.49%) and *D. seriata* (6.69%) also detected in high rates. Interestingly, the results of metagenomics and metatranscriptomics revealed a greater species complexity than suggested by fungal isolation, and also that the species profile determined by metagenomics exhibited a significant linear correlation with the results of metatranscriptomics [27,42].

Paolinelli and colleagues [37] have also used a metatranscriptomic approach to delve deeply into the etiology of the trunk disease known as *hoja de malvón* (HDM). HDM is a trunk disease particularly worrisome for Argentinean and Uruguayan vineyards that is related to esca and is commonly found in vines older than 20 years [43]. Paolinelli and colleagues carried out a metatranscriptomic study for microbiome characterization of wood from both symptomatic and asymptomatic leaves on mature field-grown *Vitis vinifera* cv. Malbec plants (Table 1). Identified bacteria represented 9.8–15% of total reads, whereas fungal reads represented 11.3–16.5%. Actinobacteria, Proteobacteria and Bacteroidetes were the most prominent bacterial phyla. In the Eukarya domain, Fungi were the most abundant kingdom, representing, on average, 14.4% of total reads. Ascomycota was the most abundant phylum, while Basidiomycota were only present at 0.8–1.0%. At the class level, Dothideomycetes (42–46%), Sordariomycetes (2–4%) and Agaricomycetes (0.2–0.4%) were the most abundant taxa. Several GTD-associated pathogens were identified, including *Diplodia*, *Neofusicoccum*, *Phaeoacremonium*, *Eutypa*, *Arambarria*, *Ilyonectria*, *Diaporthe* and *Fomitiporia*, as the most represented genera. However, different analyses carried out did not allow researchers to discriminate symptomatic from asymptomatic plants based on the presence or abundance of HDM pathogens, although alpha diversity and rank-abundance curve analyses indicated that plants with foliar symptoms had lower microbial evenness than asymptomatic plants [37]. 

In a different approach, the fungal population composition was analyzed in plants of two different lots of grafted dormant plants obtained from nurseries in Spain (cv. Garnacha Tintorera grafted onto rootstock 110 Richter) and the Czech Republic (Sauvignon Blanc grafted on SO4 rootstock) (Table 1) [28]. Plant material was divided into three different lots: an untreated control and two lots subjected to hot-water treatment (HWT) under two different conditions (50 °C/30 min and 53 °C/30 min). After the HWT plants were processed, RNA was extracted from samples corresponding to the grafting areas and the basal end of the rootstocks, which were later subjected to a metatranscriptomic analysis. Plants from the same lots were also analyzed by using traditional culture-dependent techniques for isolation and characterization of inhabiting fungi. A comparative study using different diversity estimators clearly showed that a much more diverse fungal community was revealed by the metatranscriptomic approach than by the traditional isolation approach. Thus, by the traditional isolation approach, a total of 93 fungal taxa were identified, representing 22 orders, 35 families and 67 genera. Ascomycota was the dominant fungal group, representing 15 orders, 24 families and 55 genera, followed by Basidiomycota (five orders, six families and seven genera), Mucoromycota (two orders, two families and two genera) and Zygomycota (one order, one family and three genera). However, a total OTU number of 10,585 were detected by using the metatranscriptomic approach: OTUs were assigned to 45 orders, 82 families and 189 genera. A large majority of these OTUs were Ascomycota (26 orders, 50 families and 129 genera), followed by Basidiomycota (17 orders, 33 families and 55 genera), Mucoromycota (two orders, two families and two genera) and Zygomycota (one order, one family and one genus). This study also allowed researchers to conclude that fungal communities differed in the two grapevine genotypes analyzed and that after HWT the plant material still exhibited a high fungal diversity, clearly suggesting that HWT does not sterilize the internal wood of grapevine. It is even more interesting that the fungal communities associated with GTDs changed after HWT. Among the identified taxa, 10 genera are generally regarded as being associated with GTDs: *Botryosphaeria*, *Cadophora*, *Dactylonectria*, *Diaporthe*, *Diplodia*, *Eutypa*, *Ilyonectria*, *Neofusicoccum*, *Phaeoacremonium* and *Phaeomoniella*. HWT significantly reduced all the pathogens in the Garnacha Tintorera/110 Richter combination, with the only exception of *Phaeoacremonium* spp. immediately after HWTs at 50 °C and 53 °C. In a similar way, HWT also reduced the population of GTD-associated pathogens in the Sauvignon Blanc/SO4 combination, with the exception of members of the *Diaporthe* and *Phaeoacremonium* genera, which exhibited an increase in relative abundance after HWT at 53 °C for 30 min.

## 5. The Microbiome of Soil, Rhizosphere and Root Compartments

### 5.1. The Microbiome of Vineyard Soils and Its Relationship to the Rhizosphere Microbiome

The rhizosphere is the narrow zone of soil that is in direct, intimate contact with the root and is influenced by root activity. The rhizosphere contains very high microbial populations (up to 10^11^ microbial cells per gram), thereby supporting abundant numbers of bacteria and fungi and their activity, as compared to bulk soil [44]. The microbial diversity of the rhizosphere is also very high, and in fact, there are estimations quantifying the prokaryotic diversity at more than 33,000 species per gram [45]. However, other evidence indicates that bacterial diversity in the rhizosphere is generally lower than in bulk soil [46], and microbial community composition is very different [47,48], suggesting a strongly selective environment [49]. In fact, many different factors can shape the microbial population of the rhizosphere, including ambient conditions, soil properties and especially root secretions. Plant roots release a huge variety of carbon-containing compounds known as rhizodeposits (border cells, exudates, mucilage and nutrients) which increase the nutritive character of the rhizosphere when compared to bulk soil, many of them acting as attractants for microorganisms to become established in the rhizosphere [50]. In addition, different evidences indicate that plants are able to shape their rhizosphere microbiome, as shown by the fact that different plant species host specific microbial communities when grown in the same soil [51]. 

Different studies analyzing the vineyard soil microbiome have been carried out, and their results mostly support the conclusions obtained by microbiome analysis of soils from other crops. Thus, it is well established that bacterial and fungal communities associated with bulk soil in vineyards depend on different biogeographical and edaphic factors [13,17,52], and also on different management regimes [17]. Thus, the fungal-community composition under integrated management is significantly different from that detected in vineyard soils under organic and biodynamic management, whereas fungal species’ richness remained unaffected. Moreover, studies have shown that the use of different types of green manures was a great source of biodiversity and significantly changed the microbial richness and community composition compared with other soils [19]. Interestingly, soils under integrated management had significantly reduced bacterial-species richness compared to organic soil, but community composition was similar to organically and biodynamically managed soils. The highest fungal richness was obtained under cover crops between rows in topsoil, arising from cover crop and organic carbon supplies [24]. Other studies have also shown that soil bacterial communities are strongly affected by geographical features (with geographic location, climate and topography being the most important) [53] and by vineyard management (where cover crop mix was the strongest management factor affecting soil microbiota, but tillage and compost addition were also significant factors) [17]. However, Castañeda and Barbosa [18] concluded that soil microbial communities from organic vineyards in different regions of Chile (Table 1) and the surrounding forests are quite similar, suggesting that taxonomic composition does not significantly differ between nearby habitats. The authors also suggested that native forests surrounding vineyards could act as microbial reservoirs buffering land conversion [18].

In recent years, significant advances have been achieved in deciphering the composition of microbial populations associated with the grapevine rhizosphere and how it is modeled by diverse factors, as we review here next. Zarraonaindia and colleagues [13] analyzed and compared the bacterial microbiome of bulk soil, roots and rhizospheres in five different vineyards (Suffolk County, NY, USA) (Table 1). Overall, 33–48% of belowground OTUs were shared among all belowground sample types (bulk soil, root zone soil and roots). Across vineyards, cultivars, year and plant developmental stages, the bulk soil samples had 17 OTUs in common, while the root-zone samples had 15 and roots had 10. The soils and rhizosphere samples were dominated by *Proteobacteria* (32%), with this taxon being more represented in bulk soil (up to 57%), followed by *Acidobacteria* spp. (19% in soil; 10% in root), *Bacteroidetes* spp. (10% in soil; 13% in root) and *Verrucomicrobia* spp. (8% in soil; 5% in root), with a greater relative abundance of *Planctomycetes* spp. in soil (7%) and *Actinobacteria* spp. in roots (5.1%). Sample type (bulk soil, root zone soil and root) was found to be the major explanatory variable (45% explained) of microbial community structure. Furthermore, interestingly, a multivariate regression tree that included all experimental factors (sample type, vineyard, cultivar, year and plant developmental stage) and all edaphic factors (pH, moisture, soil temperature, total C and N, and C:N ratio) identified the sample type as the most significant variable. Remarkable similarities among rhizosphere and bulk soil microbial communities were found, but the variance explained by bulk-soil versus root-zone samples was smaller than the variance explained by year, pH or vineyard. All of these results suggest that, in addition to plant selective pressure, soil structure and the rhizosphere microbiota were significantly influenced by the soil pH and C:N ratio [13]. 

Another study analyzing the bacterial microbiome of grapevine rhizosphere and roots compared to that of four weed species growing in the same vineyard (proximity of Lake Neusiedl, Austria) (Table 1) showed that the plants hosted significantly different microbiomes in their rhizospheres, and particularly in association with their roots, where differences were more pronounced in the root compartment. Again, these data strongly suggested that different plant species that are growing in the same bulk soil are able to select and recruit different microorganisms to colonize their rhizosphere environments [21]. 

The microbiome of both bulk soil and rhizosphere was also analyzed in an Italian vineyard (c.v. Pinot Noir) (Table 1) [20] under an integrated pest-management system. Biodiversity was higher in the rhizosphere than in bulk soil, independent of the phenological stage. Actinobacteria was the dominant class, with frequencies higher than 50% in all the soil samples, followed by Proteobacteria, Gemmatimonadetes and Bacteroidetes. While Actinobacteria and Proteobacteria are well-known as being dominant in soil, this was the first time the presence of Gemmatimonadetes has been observed in vineyard soils at a high rate, where it was more abundant in bulk soil (8%) than in the grapevine rhizosphere (4%). It has been reported that this phylum is one of the top nine phyla commonly found in soils, representing 0.2 to 6.5% of the bacterial diversity, with a mean of 2.2% [54]. In the conclusion, the authors indicated that the microbial-community structures differed between bulk and rhizosphere soil, and this variability was not related to the plant phenological stage [20]. 

In a similar approach, Martínez-Diz and colleagues [32] analyzed the mycobiome associated with bulk soil, rhizosphere and endorhizosphere from five different vineyards (CV. Tempranillo) in La Rioja (Spain) (Table 1). Alpha-diversity of fungal communities in soil and root samples did not differ significantly between vineyards, since 41.4% of fungal OTUs were shared among all the samples. Fungal communities were largely affected in their diversity and composition by soil-plant compartments. The endorhizosphere compartment differed most from the other two, suggesting that root tissues create a barrier for fungal colonization. The results of functional prediction suggested an increase in the relative abundances of potential plant pathogens, endophytes and arbuscular mycorrhiza, and a decrease in wood, dung and undefined saprotrophs from bulk soil toward the endorhizosphere. Roots of asymptomatic vines were a microbial niche that is inhabited by soil-borne fungi associated with grapevine trunk diseases [32].

### 5.2. The Microbiome of Grapevine Rootstocks

Since the end of the 19th century, *V. vinifera* varieties have been cultivated as scions grafted onto resistant rootstocks of other *Vitis* species and hybrids to prevent vineyards from succumbing to the devastating root phylloxera pests, as well as other pathogens. Rootstocks are also strategic in increasing *V. vinifera* productivity under harsh environmental conditions, while mining agricultural inputs (irrigation, fertilizer and pesticides) [13,55]. Moreover, rootstocks are important in preventing problems due to soil conditions, such as salinity or poor mineral nutrition [56]. It is therefore not surprising that considerable effort has been made in recent years to determine how grapevine rootstock genotypes shape the rhizosphere microbiome [23,29,31,34].

In a study carried out in vineyards in the Modena region (Italy) on Lambrusco variety grapevines grafted onto 5BB or 1103P rootstocks (Table 1), the soil bacterial microbiome was characterized in both the rhizosphere and root endosphere [23]. Clear differences were observed in OTU distributions in the grapevine root system. The authors distinguished between three groups of OTUs: the first group was defined as root (Ro_OTUs) and included all the OTUs showing a preference for (i.e., significantly more abundant in) the root ecosystem, compared to the rhizosphere and the soil; the second group, defined as root/rhizosphere (RR_OTUs), was composed of OTUs with a tropism for both root and rhizosphere (i.e., enriched in both plant compartments, compared to the soil); and, finally, the last group, named rhizosphere (Rh_OTUs), included all the OTUs significantly more abundant in the rhizosphere ecosystem than in the soil or root. The Ro_OTUs group was enriched in Proteobacteria, Bacteroidetes and Actinobacteria and showed a higher number of community-specific OTUs compared to the other groups. On the other hand, the RR_OTU and Rh_OTU groups were exclusively dominated by Proteobacteria and Actinobacteria. Interestingly, 5BB and 1103P rootstocks showed a different OTU compositional layout within the three tropic groups. In particular, members of Betaproteobacteria were included in the Ro_OTU group of 5BB only. Notably, the authors observed a progressive decrease of microbial diversity from soil to rhizosphere and root endosphere. Their data suggested that the enrichment in Actinobacteria, Bacteroidetes and Proteobacteria observed in the root endosphere-inhabiting microbiota is the result of a gated community assembled from the taxonomically congruent surrounding soil and rhizosphere biomes. In conclusion, different rootstocks used for grafting the same cultivar (i.e., the Lambrusco cultivar) select for different plant microbiotas in root endosphere and rhizosphere compartments [23].

A similar conclusion was obtained by analyzing the bacterial microbiome of Barbera variety grapevine plants grafted on 402A, 157.11, SO4 and 161.49 rootstocks in a vineyard located in Oltrepo’ Pavese (Italy) (Table 1). When the microbiomes of both rhizosphere and root tissues were analyzed, they were found to be mostly composed of Proteobacteria, Actinobacteria, Bacteroidetes, Chloroflexi and Acidobacteria phyla. However, the authors clearly concluded that bacterial communities in the root system of grape plants cultivated in the same soil and vineyard are significantly associated with the rootstock host genotype. Interestingly, despite selecting different bacterial species, grape root genotypes selected similar plant growth promoting (PGP) traits carried by different bacteria that provide fundamental ecological services [29].

More recently, Dries and colleagues [34] analyzed the bacterial microbiome associated with different grapevine rootstocks (Table 1). At the phylum level, the rhizosphere was dominated by Acidobacteriota (35%), Proteobacteria (22%), Latescibacteriota (15%), Methylomirabilota (6%) and Gemmatimonadota (4%). Several interesting findings can be highlighted from this study. Firstly, different prior studies had indicated that Proteobacteria and Actinobacteria [29,31] were the prevalent phyla in the grapevine plant rhizosphere. Both phyla play an important role in carbon cycling and production of secondary metabolites [57]. Proteobacteria is a very large and diverse phylum that includes bacteria with a broad variety of metabolic capabilities; Alpha-, Beta-, Gamma- and Delta-Proteobacteria are the classes mostly described in soil studies. However, Dries and colleagues only detected Alpha- and Gamma-Proteobacteria and in their study [34]. Both classes are considered copiotrophs (r-strategist), which are able to easily colonize environments characterized by an abundance of nutrients, such as the rhizosphere [58]. Conversely, Actinobacteria are described as oligotrophic k-strategists growing slowly and exhibiting low nutritional requirements [59]. Secondly, the number of organisms in the Acidobacteriota phylum was unusually high (~35%), and greater than that of the Proteobacteria phylum (~22%). The Acidobacteriota phylum is one of the most widespread and abundant on the planet [60], but its abundance decreases when moving from bulk soil to the rhizosphere [61]. Information about Acidobacteriota functionality is scarce, since many of its members are very difficult to isolate and cultivate in the laboratory [62]. In fact, a negative correlation between the abundance of Acidobacteria and the concentration of organic carbon in soil has been noted, which, together with their low growth rate, strongly suggests that members of this phylum are mostly oligotrophic bacteria [60]. Additionally, a high abundance of Acidobacteria, together with an unusually low abundance of Actinobacteria has been reported, suggesting that Acidobacteriota can replace Actinobacteria in the rhizosphere environment under certain conditions [13]. Third, another aspect of this study [34] that should be highlighted is the description for the first time of the phylum Latescibacterota (previously known as WS3) among the most abundant phyla in vineyard soils. Additionally, the researchers showed that phylum Gemmatimonadota organisms were also detected in significant quantities in the rhizosphere, although some previous reports indicated their presence in vineyard bulk soils in high quantities [20]. Finally, the authors of this study concluded that grapevine rootstock genotypes were associated with different rhizosphere microbiomes [34].

As mentioned above, several different studies have been carried out dealing with the analysis of bacterial microbiomes in rhizosphere microenvironments associated with different rootstocks. However, works dealing with the deciphering of fungal microbiomes are scarce. Berlanas and colleagues [31] analyzed both the fungal and bacterial microbiomes associated with different rootstocks in young and mature vineyards located in Northeastern Spain (Table 1). The vineyards were separated by 45 km and varied in most of their soil physicochemical properties. Bacterial communities of rhizosphere soil samples did not differ significantly between vineyards. Comparing the fungal and bacterial microbiota of the two vineyards, we see that 82.9% and 58.7% of bacterial and fungal OTUs, respectively, were shared between vineyards, demonstrating the existence of a “core” grape phylogeny that was independent of vineyard location. In both vineyards, Proteobacteria (26.1–28.1%) and Actinobacteria (18.5–24.1%) phyla were the most represented, comprising almost 50% of the total bacteria detected. These phyla were followed by Acidobacteria (13.7% and 16.4%), unidentified bacteria (11.4% and 11.7%) and Bacteroidetes (5.2% and 6.1%). Regarding the fungal taxa, the most abundant fungal phylum was Ascomycota (66.6–69.9%), followed by Basidiomycota (ranging from 11.5 to 20.1%) and Zygomycota (8.9% and 15.2%). Bacterial and fungal diversity in rhizosphere soil samples differed significantly among rootstocks. The results also demonstrated that bacterial microbiomes varied profoundly between years. In this study, the authors used qPCR to quantify the levels of black-foot disease pathogens in the same soil samples, in particular, those belonging to the genera *Dactylonectria*, *Ilyonectria*, *Neonectria* and *Thelonectria*. Interestingly, the authors were able to compare the relative abundance of reads with the relative abundance of black-foot disease pathogen DNA and observed a significant positive correlation. Nevertheless, they did not observe a clear correlation between known disease resistance in individual genotypes and the fungal communities, although *Cylindrocarpon*-like asexual morphs were found in lesser abundance in 161-49 C rootstocks when using both high-throughput amplicon sequencing and qPCR approaches. This rootstock had been previously recommended within an integrated management program for other grapevine trunk diseases, such as Petri disease and esca, as it appears to show a lower susceptibility to infection by certain GTD-associated pathogens [63].

## 6. The Grapevine Microbiome as a Source of More Efficient and Promising BCAs against GTDs

Since the 1990s, numerous studies have been conducted on the control of GTDs by using BCAs. More than 40 BCAs have been tested at different levels (in vitro, in planta and in the field), as reviewed by Mondello et al. [7]. Unfortunately, despite the fact that many different microorganisms have been tested, currently, only *Trichoderma* spp. have been shown to be the most suitable agents for biological control of GTDs, in both the field and nurseries, although with limited efficacy [7]. This is why researchers have recently redoubled their efforts to isolate and characterize more efficient BCAs and have turned their attention to the grapevine microbiome as a possible source for the best BCAs. The expectation is that BCAs which share the same ecological niche as the pathogen they are going to combat are showing a desirable trait for an effective BCA [64].

Fungal pathogens causing GTDs mainly enter the plant through two different routes. It is widely accepted that many pathogens are able to colonize and penetrate through the numerous pruning wounds that are produced on the plant every year [1,65,66,67]. This is especially important for those pathogens involved in Eutypa dieback, Botryosphaeria dieback, Phomopsis dieback and esca, since these diseases are primarily spread through dispersion of airborne spores [1]. Many of these pathogens can produce different types of resistance forms (ascospores, conidiospores or chlamydospores) that are able to infect the bark and/or the surface of dead grapevine wood and are able to survive for a long time in both plant material and soils in order to be later dispersed by different agents, such as air, rain or different animal vectors [1]. The second main route for infecting grapevine plants is the root system. This is the preferred route of infection by pathogens involved in black-foot disease and Petri disease, which are able to develop most of their natural cycle in soil; therefore, they can be considered soil-borne fungal pathogens [68]. Furthermore, some of these pathogens, such as *P. chlamydospora*, *P. minimum* and several members of the *Botryosphaeriaceae* family, can develop an endophytic phase, which has been isolated from the interior of both diseased and asymptomatic plants [1,5,6,69].

Next, we review (Table 2) the most recent advances relating to the characterization and effectiveness of BCAs isolated from the grapevine microbiome to prevent infection in planta through the two infection routes indicated above.

### 6.1. Actinobacteria as Promising BCAs against Soil-Borne Fungal Pathogens Causing GTDs

One promising tool for the control of GTDs caused by soil-borne fungal pathogens infecting plants through the root system is the use of Actinobacteria-based BCAs. Actinobacteria are a complex group of Gram-positive bacteria making up around 10% of the total soil microbiome [70], including soil in vineyards [13,17,18,19,20,21,24,53]. Actinobacteria are also a predominant taxa in the grapevine rhizosphere [13,20], even associated with different types of rootstocks [23,29,31,34]. They are also well-known as important secondary metabolite producers, excreting antibiotic and antifungal compounds, and for their ability to control plant diseases [71,72,73,74]. A very interesting group of Actinobacteria is that of the Streptomycetales and, particularly, the genus *Streptomyces*, which are surprisingly diverse (around 600 species). They are responsible for the production of half of all known antibiotics [74] and are well-known for their ability to control plant diseases, including soil-borne fungal pathogens [68,75,76]. 

Álvarez-Pérez and colleagues [77] isolated 58 endophytic and 94 rhizosphere Actinobacteria isolates from 1-year-old grafted plants. Based on an in vitro bioassay, 15.5% of the endophytic isolates and 30.8% of the rhizospheric isolates exhibited antifungal activity against the fungal pathogen *D. seriata*, whereas 13.8% of the endophytic isolates and 16.0% of the rhizospheric isolates showed antifungal activity against *Da. macrodidyma*. The strains which showed the greatest in vitro efficacy against both pathogens were further analyzed for their ability to inhibit the growth of *P. chlamydospora* and *Ph. minimum.* Three rhizospheric and three endophytic isolates (Table 2) were selected and applied on grafts in an open-root field nursery in a three-year trial. The field trial led to the identification of one endophytic strain, *Streptomyces* sp. VV/E1, and two rhizospheric isolates, *Streptomyces* sp. VV/R1 and *Streptomyces* sp. VV/R4, which significantly reduced the infection rate produced by the fungal pathogens *Dactylonectria* sp.–*Ilyonectria* sp. group (reduction in infection rate ranging between 66.6 and 88.9%), *P. chlamydospora* (59.4–71.1%) and *Ph. minimum* (77.8–88.9%) [77].

**Table 2:** Potential of the grapevine microbiome as a source for the isolation of BCAs against phytopathogenic fungi involved in GTDs with proven efficacy in *in plant* trials.

BCA	Source	Efficacy against GTD Pathogens in *in Plant* Trials	Reference
*Bacillus pumilus* (S32), *Paenibacillus* sp. (S19)	Wood tissue	Reduction of necrosis length caused by *P. chlamydospora* and induction of systemic resistance in planta (*B. pumilus*)	[78]
*Pythium oligandrum*	Rhizosphere	Significant reduction (40–50%) of necrosis in cv. Cabernet Sauvignon cuttings caused by *P. chlamydospora* in greenhouse assays	[79]
*Streptomyces* sp. VV/E1, VV/E2 and *S. peucetius* VV/E5	Root endophytes	Significant decrease in infection level of grapevine grafts in nursery by *Dactylonectria* sp., *Ilyonectria* sp., *P. chlamydospora* and *Ph. minimum*	[77]
*Streptomyces* sp. VV/R1, VV/R4 and VV/R5	Rhizosphere soil	Significant decrease in infection level of grapevine grafts in nursery by *Dactylonectria* sp., *Ilyonectria* sp., *P. chlamydospora* and *Ph. minimum*	[77]
*Pythium oligandrum*	Rhizosphere	Important reduction of necrosis lengths within the scion stem caused by *N. parvum* and *P. chlamydospora*	[80]
*Pantoea agglomerans* (S1); *Paenibacillus* sp. (S19)	Wood tissues (S19) or grape berries (S1)	Significant reduction of internal necrosis caused by *N. parvum* in stems	[81]
*Bacillus subtilis* PTA-271	Rhizosphere soil	Significant reduction in full dieback by *N. parvum* in Chardonnay variety. Significant protective effect observed when co-applied with fungal BCA *T. atroviride*	[82]
*Streptomyces* sp. VV/E1 + VV/R4	Root endophyte and rhizosphere soil	Very high biocontrol effect on black-foot disease pathogens *Dactylonectria torresensis* and *Da. macrodidyma* in 2-year field trials	[83]
*P. oligandrum* Po37	Rhizosphere soil	Very high biocontrol effect on *P. chlamydospora* and *P. minimum* in 2-year field trials	[83]

Molecular markers based on sequence-characterized amplified regions (SCARs) were developed for VV/E1 and VV/R4 strains. Both strains were inoculated in 1-year-old plants (cv. Tempranillo) by direct injection into the root insertion zone, or by immersion of the root system in a bacterial suspension for 24 h. The plants were then immediately planted in plastic pots, harvested 180 days after potting and analyzed to detect inoculated bacteria. Both methods allowed for effective colonization of the root system in the analyzed plants and could easily be adopted in nurseries (especially the inoculation by immersion) to produce plants colonized by BCAs [84].

*Streptomyces* sp. VV/E1 and VV/R4 strains were also tested in a comparative study, together with other bacterial and fungal BCAs [83]. Both strains were applied jointly to 1-year-old hot-water-treated plants (cv. Tempranillo) by immersion of their root systems in a bacterial suspension. After inoculation, the plants were planted in open-field vineyards to undergo a normal development. Nine (2-year-old) and 21 months (3-year-old plants) after being planted, the plants were uprooted and analyzed to search for both necrotic lesions and pathogenic fungi in the wood samples of the roots and rootstocks. The performance of both BCAs was the best of all the BCAs tested in that study against pathogens involved in black-foot disease. In fact, both BCAs were highly effective in reducing the black-foot disease incidence of *Da. torresensis* and *Da. macrodidyma* pathogens in plants of both ages. These strains also reduced the severity of infection in 2-year-old plants in the basal ends. However, the effect of these Actinobacteria against Petri disease pathogens after 2 years in the field was low. The difference in their effectiveness against Petri disease between experiments could be due to the commonly unpredictable behavior of BCAs when tested in different environments [83].

### 6.2. BCAs Based on Other Types of Bacteria

Different studies have shown the promise of the grapevine microbiome as a source of bacterial strains with the potential to be BCAs against fungal pathogens causing GTDs. Thus, Andreolli and colleagues [85] isolated endophytic bacteria from woody grapevine stems from 3- and 15-year-old plants. Bacilli and Actinobacteria were frequently isolated from 3-year-old plants, whereas Alpha- and Gamma-Proteobacteria classes were more prevalent in the 15-year-old plants. These results are in agreement with different analyses of the grapevine endomicrobiome which indicate that *Bacilli*, *Actinobacteria* and *Proteobacteria* are the predominant taxa in that environment [37]. An antifungal-activity analysis showed that two *Bacillus* strains exhibited a growth antagonistic effect toward the pathogens *N. parvum*, *P. chlamydospora* and *P. minimum*. Unfortunately, the potential of these strains has not yet been tested in in planta assays.

Additionally, 46 bacterial strains isolated from wood tissue (35) and grape surfaces (11) [86] were tested to see if they inhibit *P. chlamydospora* development [78]. In planta trials allowed for the selection of *Bacillus pumilus* (S32) and *Paenibacillus* sp. (S19) as the two most promising BCAs, since they significantly reduced the length of internal necrosis in stem cuttings (reductions ranged between 31.4 and 38.7%). Volatile compounds secreted by both strains were identified by gas chromatography/mass spectroscopy (GC/MS). The volatile compounds 1-octen-3-ol and 2,5-dimethyl pyrazine were obtained commercially and tested, showing strong antifungal activity against *P. chlamydospora*, suggesting that these compounds may play an important role in the bacterial antagonistic activity in planta. The authors also demonstrated that *B. pumilus* (S32) induced systemic resistance in grapevine. Therefore, it can be concluded that both BCAs can control *P. chlamydospora* infection via direct and/or indirect mechanisms [78]. In a more recent study [81], *Paenibacillus* sp. (S19) and *Pantoea agglomerans* (S1) (Table 2) were tested to control *N. parvum* in assays developed in potted plants via application of BCAs in both cutting-stem assays or directly to the soil. This last strain had been isolated from the surface of grape berries [86]. The inhibitory activity of both strains against *N. parvum* was significantly dependent on the application method. Thus, application to stems was much more efficient than soil inoculation. When performing preventive inoculation on stems, the inhibition of *N. parvum* wood necrosis reached 50% and 65% for *P. agglomerans* (S1) and *Paenibacillus* sp. (S19), respectively. The mechanisms of action responsible for the inhibitory effect were different, since *P. agglomerans* (S1) inhibited *N. parvum* via the secretion of antifungal volatile compounds, while *Paenibacillus* sp. (S19) mainly inhibited this pathogen via antibiosis. In addition, both BCAs induced systemic defenses in grapevine [81]. Interestingly, it should be noted that *P. agglomerans* was able to significantly combat the pathogen inside vine stems, although, as indicated above, it was isolated from grape surfaces. These data suggest that some BCAs isolated from a certain niche of grapevine plants may be able to function effectively and colonize other niches in the plant.

*Bacillus* could be one of the most interesting genera as a source of BCAS for controlling GTDs, as shown in other studies. Thus, a *B. subtilis* F62 strain has shown some effectiveness against *Da. macrodidyma* (involved in black foot disease) when inoculated on grapevine plants. This strain is defined by the authors as a soil-borne strain, but no data regarding a putative isolation from grapevine microbiome are indicated [87]. However, the *B. subtilis* PTA-271 strain was isolated from the rhizosphere of healthy Chardonnay grapevines from a vineyard located in the Champagne area (France) [82] (Table 2). This bacterial BCA, alone or in combination with *Trichoderma atroviride* SC1 (Vintec^®^, Belchim Crop Protection), a BCA isolated from decaying wood of hazelnut tree [88], has been tested in in planta analysis against the pathogen *N. parvum*. The BCAs were applied to plants in different ways: whereas *B. subtilis* was applied to the soil, *T. atroviride* was applied to wounds made in stems. *B. subtilis* was able to significantly reduce (45%) the number of plants with full dieback in the Chardonnay variety, whereas no reduction was observed when *T. atroviride* was applied. Results in the Tempranillo variety were the opposite. The fungal BCA significantly reduced full dieback, whereas the bacterial BCA was unable to provide effective protection. These data suggest that the beneficial effects of the BCAs tested are cultivar-dependent. Interestingly, when both BCAs were combined, an effective protection was obtained for the Tempranillo variety, whereas this protective effect was not observed in the Chardonnay variety. Plant systemic immunity was also affected by each BCA. The authors suggest a common feature for the two cultivars: the defenses that are greatly diminished in BCA-protected plants appear to be those that are responsive to salicylic acid (SA), in contrast to symptomatic plants. For Tempranillo, the high basal expression of SA-dependent defenses may thus explain the highest susceptibility to Botryosphaeria dieback and also the ineffectiveness of *B. subtilis* PTA-271 under the experimental conditions tested [82].

Recently, Niem et al. [33] identified several *Pseudomonas* isolates colonizing the wood interior that exhibit good antifungal activity in vitro against several GTA-associated pathogens, but, unfortunately, their in planta effectiveness has not been tested yet.

### 6.3. BCAs Based on Rhizospheric or Endophytic Fungi 

Several recent studies have pointed to an interest in endophytic fungi as putative BCAs against GTD pathogens [89,90], but, unfortunately, there have not yet been any trials in plants to test their effectiveness. One of the most promising fungal BCAs against GTDs is the oomycete *Pythium oligandrum*, which is a frequent inhabitant of the grapevine root system from which different isolates have been obtained [80,91]. Different strains have been able to significantly reduce (40–50%) the necrosis of *V. vinifera* (cv. Cabernet Sauvignon) cuttings caused by *P. chlamydospora* in greenhouse assays when *P. oligandrum* colonized the plant root systems. The BCA was able to induce plant resistance to the pathogens by increasing expression levels of different genes involved in various pathways (PR proteins, phenylpropanoid pathways, oxylipin and Redox systems) [79]. This oomycete exhibited a good antifungal performance against two major fungal pathogens, *N. parvum* and *P. chlamydospora*. By considering the reduction in necrosis lengths within the scion stem, treatments with *P. oligandrum* alone showed the greatest efficacy against the two pathogens (overall average efficacy of 48.3%) [80]. This BCA was jointly applied with two different bacterial BCAs (*P. agglomerans* and *B. pumilus*), but, unfortunately, a synergistic effect for both kinds of BCAs was not observed. In a different trial, *P. oligandrum* Po37 was able to significantly reduce *P. chlamydospora* and *P. minimum* infections [83]. Finally, *P. oligandrum* is also able to induce defense mechanisms against the trunk pathogen *N. parvum* [92]. 

## 7. Conclusions

In the last few years, several different analyses of the grapevine microbiomes have been carried out. These studies indicate that vineyard soil is a microbial reservoir whose composition is strongly influenced by numerous factors, such biogeographical aspects, physicochemical properties, climatic conditions or type of vineyard management (conventional or organic), to mention only the most relevant ones. As a microbial reservoir, soil shares many microbial taxa with the microbial communities of the different plant niches (root environment, canes, cordons, flowers, leaves and berries). However, as we analyze the microbiota of plant compartments, we observe how microbial diversity decreases in relation to that of the soil. This is especially remarkable in the root system environment, where microbial diversity of the rhizosphere is lower than that of the soil, and, at the same time, microbial diversity in the rhizosphere is higher than that of endophytic populations. Grapevine plants obviously exert a strong selection on the microorganisms that will occupy each niche in the plant, and this is especially relevant at the rhizosphere level, where we can even detect striking differences for different rootstocks in a same soil.

Many of the most abundant bacterial taxa in all the plant compartments (phyllosphere, reproductive organs, woody parts and the root system), such as Bacillales (*Bacillus* sp. and *Paenibacillus* sp. isolates), and Actinobacteria (*Streptomyces* sp. isolates), which are especially abundant in the root environment and the endosphere, have been proven to be a promising source of recently developed bacterial BCAs to combat GTDs. Moreover, the Enterobacteriales order (*Pantoea* sp.), which is well represented in the phyllosphere, may contribute to increasing the arsenal of BCAS against GTDs.

The grapevine plant mycobiome also represents an interesting source of fungal BCAs, with the case of *Pythium oligandrum* being especially relevant. Recent reports indicate that the grapevine endosphere could also provide additional fungal BCAs, although in planta trials are required in order to prove their effectiveness.

Unfortunately, although all of these BCAs have proven their effectiveness in controlling one or another GTD, we still do not have effective BCAs against a wide range of pathologies. Thus, some future aspects that remain to be addressed would be whether the combination of two or more BCAs could provide a synergistic effect that would increase the spectrum of action of the possible combinations. Furthermore, the development of new application methods, optimization of the timing of application and development of different formulations could contribute to increasing the effectiveness of these new BCAs.

## Data Availability

Not applicable.
